# Crystallins Are Regulated Biomarkers for Monitoring Topical Therapy of Glaucomatous Optic Neuropathy

**DOI:** 10.1371/journal.pone.0049730

**Published:** 2013-02-26

**Authors:** Verena Prokosch, Maurice Schallenberg, Solon Thanos

**Affiliations:** 1 Institute of Experimental Ophthalmology, School of Medicine, University of Münster, Albert-Schweitzer-Campus 1, Münster, Germany; 2 Interdisciplinary Center for Clinical Research, Albert-Schweitzer-Campus 1, Münster, Germany; University of Regensburg, Germany

## Abstract

Optic nerve atrophy caused by abnormal intraocular pressure (IOP) remains the most common cause of irreversible loss of vision worldwide. The aim of this study was to determine whether topically applied IOP-lowering eye drugs affect retinal ganglion cells (RGCs) and retinal metabolism in a rat model of optic neuropathy. IOP was elevated through cauterization of episcleral veins, and then lowered either by the daily topical application of timolol, timolol/travoprost, timolol/dorzolamide, or timolol/brimonidine, or surgically with sectorial iridectomy. RGCs were retrogradely labeled 4 days prior to enucleation, and counted. Two-dimensional polyacrylamide gel electrophoresis (2D-PAGE), matrix-assisted laser desorption ionization mass spectrometry, Western blotting, and immunohistochemistry allowed the identification of IOP-dependent proteomic changes. Genomic changes were scrutinized using microarrays and qRT-PCR. The significant increase in IOP induced by episcleral vein cauterization that persisted until 8 weeks of follow-up in control animals (*p*<0.05) was effectively lowered by the eye drops (*p*<0.05). As anticipated, the number of RGCs decreased significantly following 8 weeks of elevated IOP (*p*<0.05), while treatment with combination compounds markedly improved RGC survival (*p*<0.05). 2D-PAGE and Western blot analyses revealed an IOP-dependent expression of crystallin cry-βb2. Microarray and qRT-PCR analyses verified the results at the mRNA level. IHC demonstrated that crystallins were expressed mainly in the ganglion cell layer. The data suggest that IOP and either topically applied antiglaucomatous drugs influence crystallin expression within the retina. Neuronal crystallins are thus suitable biomarkers for monitoring the progression of neuropathy and evaluating any neuroprotective effects.

## Introduction

Elevated intraocular pressure (IOP) is a major risk factor in glaucomatous optic neuropathy [1] for review and the mainstay of glaucoma treatment continues to be lowering of the IOP by pharmacological or surgical methods [2]. However, retinal ganglion cell (RGC) loss and damage to the optic nerve may continue despite significant reductions in IOP [3]–[11].

It has been assumed that inflammatory and metabolic processes are involved in glaucomatous neuron death. Crystallins, which belong to the family of small heat shock proteins (HSPs) and comprise three major families (α, β, and γ crystallins), have been found within RGCs [12], [13]. Both neuroregenerative [13] and neurodegenerative [14] properties have been attributed to retinal crystallins. Specific regulation of crystallins has been observed in the context of neurodegenerative diseases such as glaucoma [12], [15]. Furthermore, human glaucoma patients exhibit increased titers of antibodies against small HSPs [14], [16]–[17]. Crystallins may act as critical modulators in glaucoma and thus be integral to the process of glaucomatous neurodegeneration [14].

The retina and the optic nerve provide an easily accessible and relevant model with which to study central nervous system injury and postinjury repair. Experimental, genetic, and hereditary mutant animal models of glaucoma provide suitable tools with which to study the complex process of neuronal degeneration in glaucoma [18]–[20]. We hypothesized that IOP elevation causes alterations in gene and protein expressions within retinal cells. Based on this hypothesis, different drugs may alter the expressions of such molecules, which can be analyzed by two-dimensional gel electrophoresis (2DE) and matrix-assisted laser desorption ionization (MALDI) mass spectrometry (MS) in order to identify disease- or treatment-associated proteins. In addition, proteomic–genomic correlations may help to identify novel pharmacological targets [21], [22].

Several families of drugs that aim at limiting the risk for retinal and optic nerve neuropathy are in clinical use, and all of them are designed to normalize IOP. In addition to surgical procedures, α-2a [23] and β-adrenergic [24] receptor agonists, prostaglandin F2α analogues [25], and carbonic anhydrase inhibitors [26] are the most important classes of drugs used in this context [27]. Some of these drugs are suspected to act neuroprotectively by altering retinal protein metabolism and activating signaling cascades in favor of RGC survival.

The purpose of the present study was to identify metabolic retinal changes at the genomic and proteomic levels using 2DE, MALDI-MS, microarray analysis, quantitative real-time polymerase chain reaction (qRT-PCR), Western blotting (WB), and immunohistochemistry (IHC).

## Methods

### Animals and drugs

Ethical statement and animals: All experiments were conducted in accordance with the Association for Research in Vision and Ophthalmology (ARVO) Statement on the Use of Animals in Ophthalmic and Vision Research. Sprague-Dawley rats were housed in a standard animal room under a 12-h light/dark cycle with food and water provided *ad libitum*. The ethics committee (Bezirksregierung Münster, i.e. regional government of Münster) specially approved this study (Permission Nr.: 84-02.04.2011.A132). Animals were housed in a standard animal facility with food and water *ad libitum* and a 12 hrs light-dark cycle. Surgical procedures were performed unilaterally, on the left eye of rats weighing 180–250 g, under general anesthesia induced by a mixture of 2 mg/kg body weight ketamine and 2 mg/kg body weight xylazine (Ceva-Sanofi, Düsseldorf, Germany), administered intraperitoneally. After each surgical intervention, gentamicin eye ointment (Gentamytrex, Dr. Mann Pharma, Berlin, Germany) was applied topically. The animals' health and behavior were monitored postoperatively at regular intervals. The experimental follow-up after cauterization lasted 8 weeks. Each experimental group comprised 9 animals, except for the normotensive and hypertensive groups, which each comprised 18 animals.

### Induction of glaucoma and intraocular-pressure measurement

**Figure 1:**
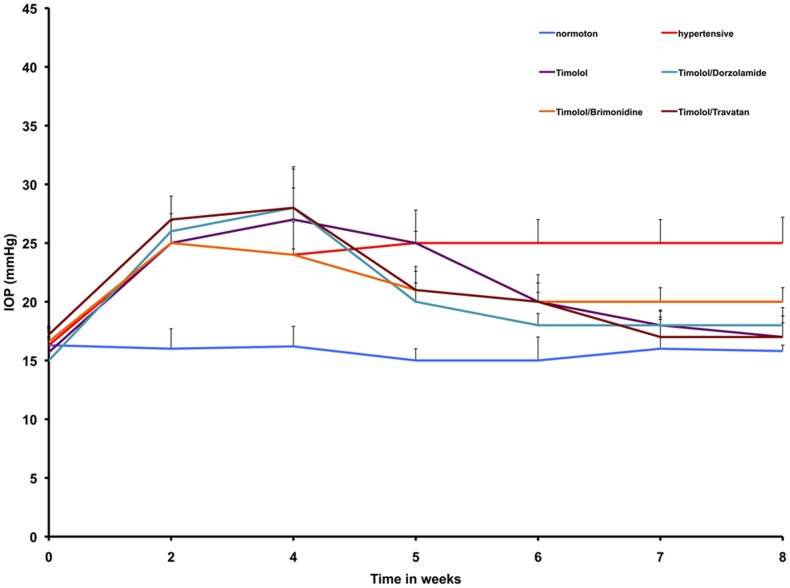
IOP readings in an experimental model of ocular hypertension. A significant increase in IOP (*p<0.05) was observed in eyes after cauterization of three episcleral veins in all groups relative to the normal control group (blue line) after 2 weeks. After the initiation of a medical hypotensive treatment, marked by a black arrow, the IOP was reduced significantly (**p<0.05) in all groups, whereas it remained elevated throughout the experimental period in the control group without hypotensive treatment (red line).

IOP was elevated through thermic cauterization of three episcleral veins as follows. The limbus-draining veins travel close to the sclera from the limbus backwards and anastomose at the equator of the eye, and were exposed by incision of the conjunctiva where they form four to five major venous trunks almost equidistant around the circumference of the globe. Ophthalmic cautery was applied to three of these large veins per eye, resulting in blockage of more than 50% of the venous outflow (Fig. 1A) [4]. Care was taken not to damage the sclera during this procedure. IOP measurements were made before and immediately after cauterization, and then every week between 9.00 a.m. and 12.00 a.m. under light anesthesia with isoflurane (Isofluran DeltaSelect, Actavis, Langenfeld, Germany) and topically applied 0.5% proparacaine (URSA-Pharm, Saarbrücken, Germany). Ten tonometer readings were taken directly from the instrument display for each eye measurement, recorded, and averaged. “Off” (or outlier) readings and instrument-generated averages were ignored. An uncauterized group (*n* = 18) served as the normotensive control. The IOP appeared to have increased 10 days after cauterization, and it remained elevated for the entire duration of the experiment. Animals in which the IOP returned to normal levels were excluded from the study. None of the animals exhibited an enlarged globe or edematous cornea. Four weeks after the IOP elevation, the hypotensive treatment was begun either surgically by iridectomy or by daily application of topical IOP-lowering eye drops for the subsequent 4 weeks of follow-up. One group of animals (*n* = 18) remained untreated, with a persistent elevated IOP; this group served as the corresponding hypertensive control.

### Application of eye drops

Eye drops containing either only 0.5% timolol (Ti, *n* = 9; 0.5% TimOphtal; Winzer Pharma, Berlin, Germany), or combinations of 0.5% T and dorzolamide (Ti/D, *n* = 7; Cosopt, Chibret, München, Germany), 0.5% T and travoprost (Ti/Tr, *n* = 7; Duotrav, Alcon, Hünenberg, Switzerland), or 0.5% T and brimonidine (TiB, *n* = 9; Combigan, Allergan, Irvine, CA, USA) were applied topically to the left eye daily between 8.00 a.m. and 10.00 a.m. over a 4-week period. Each drop was kept in situ for at least 30 s by manually holding the eye open.

### Retrograde labeling and quantification of retinal ganglion cells

Four days prior to enucleation of the eye, three rats from each experimental group were anesthetized and their RGCs retrogradely labeled from the superior colliculus (SC) using the fluorescent dye hydroxystilbamidine methanesulfonate (5% FluoroGold (5-FG)] in phosphate-buffered saline (PBS; Invitrogen, Eugene, OR, USA). This results in the exclusive labeling of RGCs in a uniform manner across the entire retina, thus enabling their quantification on retinal flat-mounts [28]. Briefly, after surgical exposure, a few solid crystals of 5-FG were inserted into the superficial layers of the contralateral right SC. The cortical cavity was filled with Gelfoam (Pharmacia and Upjohn, Kalamazoo, MI, USA) and the skin wound was sutured. The animals were subsequently allowed to survive for 4 days to allow the dye to be taken up by the axon terminals of the RGCs in the SC and transported retrogradely to the RGC somata in the retina.

Animals were killed under a carbon dioxide atmosphere, and their retinas isolated, flat-mounted, and fixed in 4% paraformaldehyde overnight at 4°C. RGCs were visualized with the aid of a fluorescence microscope (Axiophot, Carl Zeiss, Oberkochen, Germany) using a 360-nm excitation filter and a 460-nm band-pass emission filter. Images of five areas at five different eccentricities (uniform central to peripheral distribution) were obtained in each retinal quadrant at a final magnification of ×200. The optic disc served as the point of reference. RGCs were counted in a 150-µm^2^ area in each image. The number of RGCs per square millimeter was determined and averaged for each group.

### Two-dimensional gel electrophoresis and matrix-assisted laser desorption ionization mass spectrometry

2DE and MALDI-MS were performed on retinal samples from normotensive and hypertensive retinas modified with and without hypotensive treatment. 2DE was conducted using a method initially described by O'Farrell [29]. Retinal explants were harvested and boiled in 10% sodium dodecylsulfate (SDS; Sigma, Taufkirchen, Germany) and homogenized in 2DE lysis buffer (7 M urea and 2 M thiourea; Merck, Darmstadt, Germany), 4% 3-[(3-cholamidopropyl)-dimethylammonio]-1-propane sulfonate (USB, Cleveland, OH, USA), 40 mM Tris base (Carl Roth, Karlsruhe, Germany), 1 mM phenylmethylsulfonyl fluoride (Sigma), and 10 mM dithiothreitol (DTT; Roche, Mannheim, Germany). The final SDS concentration was 0.25%. Soluble protein (200 µg according to the Bradford test) together with 2% immobilized pH-gradient (IPG) buffer (pH 3–10; Amersham Biosciences Europe, Freiburg, Germany) and 20 mM DTT were loaded onto Immobiline DryStrips (pH 3–10, 18 cm; Amersham Biosciences Europe) and rehydrated overnight. The rehydrated strips were focused on a Multiphor II system (Amersham Biosciences Europe) at approximately 80 kVh. Focused IPG strips were incubated twice for 15 min in equilibration solution [50 mM Tris HCl (pH 8.8), 6 M urea, 30% glycerol, 2% w/v SDS, and a trace of bromophenol blue (Merck)], with 1% β-mercaptoethanol and 2.5% iodoacetacetamide added to the first and second equilibration steps, respectively. For the second dimension, the equilibrated IPG strips were fixed with 0.5% w/v melted agarose (Merck) on homogeneous 12.5% SDS gels (Rotiphorese Gel 30, Carl Roth). Proteins were separated by vertical SDS–polyacrylamide gel electrophoresis (SDS-PAGE; BioRad, München, Germany) according to Laemmli [30]. Protein spots were initially labeled with colloidal Coomassie Blue 250 (Merck). Spots were manually excised, tryptically digested in the gel, extracted, purified using Zigtips (microbed C18, Millipore, Bedford, MA, USA), and then subjected to MS. Peptide maps were generated using TOF-Spec-2E (Micromass, Manchester, UK), and selected retinal peptides were sequenced using nano-high-performance liquid chromatography MS/MS (Ultimate, LC Packings, Amsterdam, The Netherlands; Esquire 3000, Bruker Daltonics, Bremen, Germany). Three gel replicates were compared. National Center for Biotechnology Information and SWISS-PROT databases were searched using Mascot software (Matrix Science, London, UK). Additional image analyses were performed on gels stained with silver nitrate.

### Western blotting

The eyes of normotensive rats with elevated IOP and those subsequently treated with Ti or Ti/D, Ti/B, or Ti/Tr were enucleated, the retina was isolated, embedded in Tissue-Tek (Sakura-Finetek, Torrance, CA, USA), and frozen in liquid nitrogen. The probes were homogenized in SDS sample buffer (62.5 mM Tris HCl, 2% w/v SDS, 10% glycerol, 50 mM DTT, and 0.01% w/v bromophenol blue). After sonicating and heating the sample, the protein concentration was determined using Bradford reagents. Fifty micrograms of protein from each sample was fractionated on 8%, 10%, or 12% SDS-PAGE (depending on the examined protein) with a protein marker (BioRad, San Diego, CA, USA). After electrophoresis, proteins were transferred to a nitrocellulose membrane. The blots were incubated in blocking solution (5% fat-free dry milk and 0.1% Tween-20 in PBS) for 1 h, followed by incubation overnight at 4°C with polyclonal antigoat βb2 crystallin (cryβb2; Santa Cruz Biotechnology, Santa Cruz, CA, USA) used at a dilution of 1∶700. The polyclonal antigoat βb3 crystallin (cryβb3; Santa Cruz Biotechnology), the polyclonal antisheep βH crystallin (cryβH; Biogenesis, New Fields UK), the polyclonal antisheep βL crystallin (cryβL; Biogenesis), and μ crystallin (cryμ; Sigma-Aldrich, München, Germany) antibodies were used at dilutions of 1∶700, 1∶1000, 1∶600, and 1∶1000, respectively. Polyclonal antigoat HSP-90 (Santa Cruz Biotechnology), polyclonal antirabbit HSP-70 (Cell Signaling, Boston, MA, USA), polyclonal antirabbit HSP-25 (Upstate Biotechnology, Lake Placid, NY, USA), and polyclonal antirabbit γ crystallin (cryγ; Santa Cruz Biotechnology) antibodies were used at dilutions of 1∶1000, 1∶1000, 1∶10,000, and 1∶200, respectively. The applied control antibodies, anticalnexin (Sigma-Aldrich), antiactin (Sigma-Aldrich), and anti-GAPDH (Sigma-Aldrich), were used at a dilution of 1∶10,000. The membrane was then incubated with the secondary antibody conjugated with horseradish peroxidase in blocking solution for 1 h at room temperature. Antibody detection was performed with enhanced chemiluminescence (Amersham Biosciences, Rockville, MD, USA). The relative densities of the protein spots were analyzed using Alpha Ease (Alpha Ease FC software 4.0, Alpha Innotech, Biozym Scientific, Vienna, Austria). The protein density of a fixed area was determined for each spot after subtracting the specific background density of the same area. The spot density was correlated and normalized to the relative density of the particular application control. The normotensive spot density was defined as the reference mark, and the relative relationships were determined and processed.

### Immunohistochemistry

Frozen 10-µm-thick sections of isolated retina samples obtained from normotensive eyes and hypertensive eyes after IOP elevation were fixed in cold acetone for 10 min. They were washed three times for 5 min each in PBS and blocked with 10% fetal calf serum (FCS) for 30 min. The sections were then incubated overnight at 4°C with a primary antibody, polyclonal anti-rabbit β crystallin (gift from the Department of Biochemistry, Hyderabad, India), which was diluted at 1∶400 in 10% FCS. After rinsing the slides three times each in PBS for 5 min, the sections were incubated with the secondary anti-rabbit Cy2 antibody (Dianova, Hamburg, Germany) diluted at 1∶200 in 10% FCS for 30 min at room temperature, and then washed three times for 5 min each in PBS. Finally, the slides were coverslipped with Mowiol (Höchst, Frankfurt, Germany). The nuclei of retinal cells were stained by adding 4′,6-diamino-2-phenylindole dihydrochloride hydrate (Sigma-Aldrich) to the Mowiol embedding medium. Slides were examined with the aid of a fluorescence microscope (Axiophot, Carl Zeiss) with the appropriate filters. Negative controls comprised sections processed without addition of the primary antibodies. Control and experimental sections were stained simultaneously to avoid variations in immunohistochemical staining.

### Microarrays

Retinal samples obtained from normotensive (*n* = 3) and hypertensive (*n* = 3) rats 4 weeks after IOP elevation were used for the microarray analysis. Animals were killed in a carbon dioxide chamber, and their eyes were immediately enucleated and placed on ice. The retina was removed quickly and collected in RLT buffer, a component of the RNeasy kit (Qiagen, Hilden, Germany). A minimum of 10 µg of total RNA/retina was isolated using the RNeasy kit using the procedure described in the manufacturer's instructions. Total RNA was then shipped on dry ice to MWG Biotech (Ebersberg, Germany), where an aliquot of the RNA was subjected to quality analysis using the 2100 Bioanalyzer system. The RNA was then amplified with T7 polymerase following reverse transcription into cDNA, during which fluorescence-labeled nucleotides (Cy3/Cy5) were incorporated. The labeled probes were hybridized to 10 k chips (MWG Biotech). Three separate hybridizations per group were carried out with cDNA derived from three separate animals. The 10-k chip consists of 9715 rat genes (5535 Rat 5 k genes) spotted onto one array with an additional 4180 annotated open reading frames from an in-house MWG Biotech expressed sequence tag sequencing project.

To design microarrays with optimal hybridization conditions, existing databases are filtered for redundant sequences and the oligonucleotides are designed with the Oligos-4-Array (developed by MWG Biotech). This requires that nontarget genes be less than 75% similar over a 50-base target region. In fact, if the 50-base target region is marginally similar (50–75%), it must not include a stretch of complementary sequence of >15 contiguous bases. The oligonucleotide design thus guarantees the exclusion of both dimer and secondary structure formation. Cross-hybridization is minimized by exhaustive BLAST and global Smith-Waterman searches. The microarrays were scanned at a resolution of 10 µm at three photomultiplier gain settings in order to optimize the dynamic range. The resulting three images were integrated into one intensity value for each spot using the software packages ImaGene and GeneSight (MWG Biotech), and MAVI (MWG Biotech).

The fluorescent signals were corrected and normalized for the difference between Cy3 and Cy5. Samples from each of the three cohybridizations were compared independently of each other. The signal values of probe sets that were reliably detected in most of the experiments in each group were used in two-sample, two-tailed *t*-tests between the “experimental” and “control” groups (nonglaucomatous vs. glaucomatous retina). Probe sets were selected from candidate genes using a *t*-test based on *p*<0.05, and the ratio of means (relative change) between the two groups was calculated with “control” as the denominator. The final relative changes quoted here are the average values of three independent experiments. The cut-off values for up- and down-regulation were set at >3.0-fold and <0.3-fold, respectively. The biological function of differentially expressed genes with a change of >3.0-fold or <0.3-fold were modeled according to their biological process using the Protein ANalysis THrough Evolutionary Relationships (PANTHER) classification system (Applied Biosystems, San Diego, CA, USA). The PANTHER classification system allows high-throughput analysis of proteins (and their genes), which can be classified according to families and subfamilies, molecular functions, biological processes, and pathways.

### Quantitative real-time polymerase chain reaction

The real-time PCR was implemented in an ABI PRISM 7900 sequence detector (Applied Biosystems) in 384-well plates. For the qRT-PCR, total RNA was isolated from the retinas of a second set of animals, because there was insufficient RNA from the microarray experiments for both experiments. Five rats with IOP elevation and five animals with a normal IOP were used for qRT-PCR experiments. One microgram of total RNA was first reverse transcribed using the Omniscript Reverse Transcriptase (5 mM dNTPs, 10×RT buffer, 10 units/µl RNase inhibitor, and 10 µM Oligo-dT primer; MWG Biotech) in a total volume of 20 µl for 1 h at 37°C. The enzyme was inactivated by heating at 95°C for 5 min. The cDNA was diluted twofold, and a 1-µl aliquot was used for each 20-µl PCR using the TaqMan Universal PCR Master Mix and Assays-on-Demand (Applied Biosystems). Assays-on-Demand gene expression products consisted of a 20× mix of unlabeled PCR primers and a TaqMan MGB probe (labeled with FAM-TAMRA dye), and they were used to quantify the expression of seven genes: αA crystallin (cryαA), αB crystallin (cryαB), βB1 crystallin (cryβb1), cryβb2, cryβb3, and βA4 crystallin (cryβb4), with the 18S RNA (Assay Hs99999901_s1) gene serving as an endogenous control. The assays are designed for the detection and quantitation of specific rat genetic sequences in RNA samples converted to cDNA. The reaction components consisted of 10 µl of TaqMan Universal PCR Master Mix, AmpErase uracil-*N*-glycosylase (UNG; 2×), 1 µl of Assay-on-Demand (20×), and 1 µl of cDNA in a 20-µl reaction. The PCR conditions for all genes were as follows: UNG activation, 50°C for 2 min; preheating, 95°C for 10 min; then 40 cycles of denaturation (95°C for 15 s) and annealing/elongation (60°C for 1 min). Each sample was run in duplicate.

The data were analyzed using SDS 2.2 software (Applied Biosystems). 18S RNA served as the endogenous control against which to normalize the amount of cDNA added to each reaction (ΔCt), and the mean ΔCt of control samples was used as the calibrator to calculate ΔΔCt. The comparative Ct method was employed, whereby the relative quantity of the respective target gene mRNA—normalized to the endogenous control and relative to the calibrator—is expressed as the relative change: 2–ΔΔCt.

### Statistical analysis

All data regarding IOP recordings, RGC densities of retinal whole-mounts, and relative protein densities in WBs are presented as mean±SD values. Data were analyzed statistically using the two-independent-samples test (SPPS, Statistica version 7) for Gaussian distributions, with the remaining quantitative data analyzed using two-way analysis of variance (Statistica version 7) with post-hoc analyses using the Tukey HSD test to identify possible differences among the experimental groups. If the distribution was not Gaussian, the Kruskal-Wallis *H* test was used.

## Results

### Pharmacological effects on intraocular pressure

The baseline IOP in the normotensive sham-treated group was 15.8±1.5 mmHg. By 10–12 days after episcleral vein cauterization, the IOP had increased significantly by 1.6-fold to 24.8±1.7 mmHg (*p*<0.001). These values are consistent with those obtained by other groups, and are nearly identical to those recorded in humans, rabbits, and anesthetized monkeys [31]. The recordings were sustained for the entire duration of the experimental period if animals remained untreated. If treated hypotensively, IOP was reduced effectively as follows (*p*<0.05):

1. Ti lowered IOP to 20.00±1.65 mmHg (*p*<0.05).
2. Ti/B reduced IOP to 20.5±1.4 mmHg (*p*<0.03).
3. Ti/D and Ti/Tr produced more distinctive reductions in IOP (18.50±1.35 and 18.75±1.80 mmHg, respectively; *p*<0.001).

These recorded readings remained constant over the 4 weeks of antihypertensive treatment. IOP recordings are illustrated in detail in Fig. 1.

### Quantification of retinal ganglion cells

**Figure 2:**
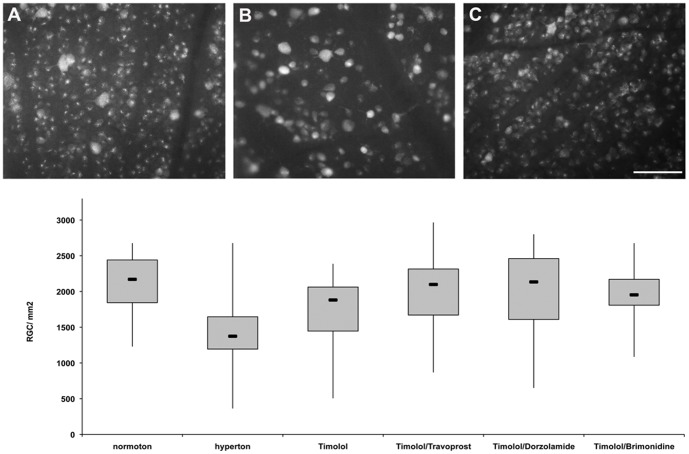
Densities of retinal ganglion cells (RGCs) labeled with 5% FluoroGold 8 weeks after the induction of elevated IOP. A RGCs in a normotensive, sham-treated retina. **B** RGCs in an untreated retina with elevated IOP without hypotensive treatment. **C** RGCs in a retina with elevated IOP treated with 0.5% timolol (Ti) and travoprost (Ti/Tr) for 4 weeks [the results were the same for those treated with 0.5% Ti and dorzolamide (Ti/D), and 0.5% Ti and brimonidine (Ti/B)]. **D** Boxplots illustrating the total RGC survival within the different experimental groups. IOP elevation significantly decreased the number of RGCs (*p<0.05) compared with the normotensive, sham-treated group, while 4 weeks of treatment with hypotensive eye drops (i.e., with Ti/Tr, Ti/D, or Ti/B), which began 4 weeks after IOP elevation, significantly improved RGC survival (**p<0.05) relative to the untreated hypertensive group. Treatment with Ti (0.5%) alone did not substantially improve RGC survival.

The density of RGCs in retinal whole-mounts of normotensive animals (*n* = 3) was 2112±369 RGCs/mm^2^. IOP elevation (*n* = 3) significantly reduced the RGC density to 1488±532 RGCs/mm^2^ (*p*<0.001) at 8 weeks after cauterization. These data are in agreement with previous RGC quantifications in glaucoma using 5-FG. Topical treatment with the combination compounds Ti/Tr, Ti/D, and Ti/B strongly enhanced RGC survival, preserving 2020±548 RGCs/mm^2^ (*p*<0.001; *n* = 3), 2031±734 RGCs/mm^2^ (*p*<0.004; *n* = 3), and 1956±340 RGCs/mm^2^ (*p*<0.001; *n* = 3), respectively. The RGC densities in the experimental groups are illustrated in Fig. 2.

### Retinal protein profiling

**Figure 3:**
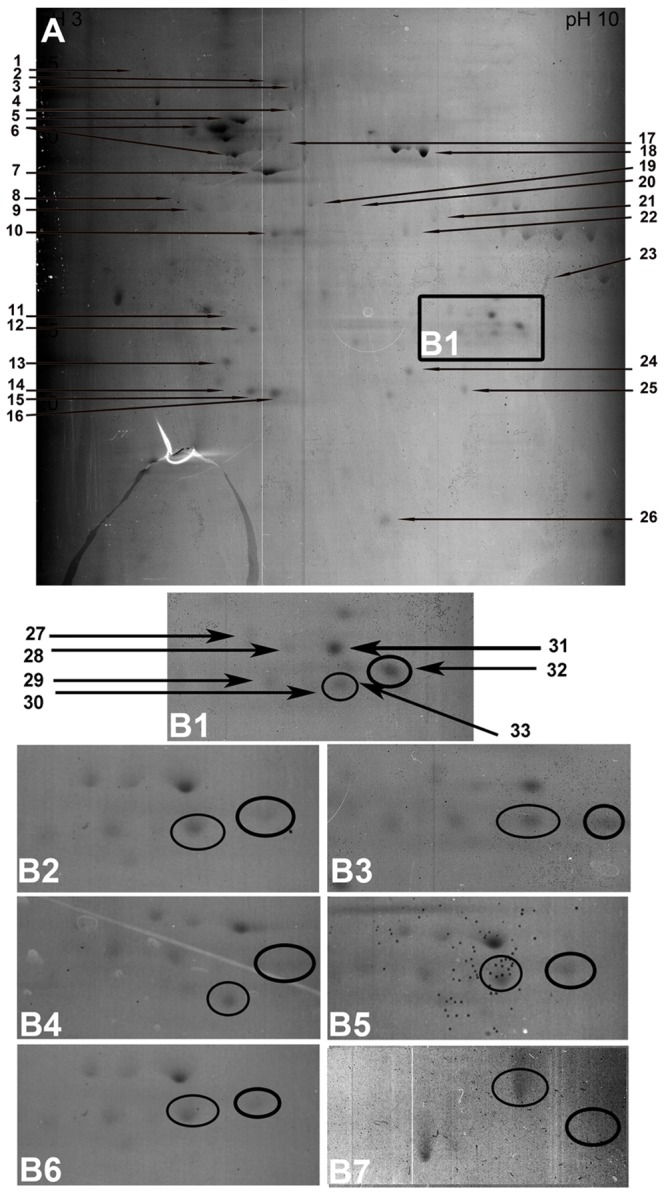
Two-dimensional gelelectrophoresis of retinal proteins. A Peptide mapping of a retina obtained from a Sprague-Dawley rat with untreated, sustained elevated IOP 8 weeks after IOP induction. Hypertensive retinal samples showed a marked increase in βb2-crystallin (cryβb2) expression (area marked by a black box in **A**, shown at higher magnification in **B1** compared to a normotensive sham-treated retina, **B2**). The 4 weeks of IOP-lowering treatment with topical treatment with Ti (**B3,B4**), Ti/T (**B5**), Ti/D (**B6**), and Ti/B (**B7**) decreased cryβb2 expression to baseline levels. The proteins identified, which are marked by an arrow in **A** and **B1**, are listed in Table 1 according to the number given.

Several protein spots were reproducibly detected with 2DE (those for the hypertensive group are shown in Fig. 3A). Landmark protein spots that appeared with consistent staining intensities in all experimental groups were first mapped and identified (listed in Table 1). In addition, a conspicuous group of proteins appeared in the middle range of molecular masses (20–30 kDa) at slightly basic pH values (Fig. 3A). This area (within the rectangular frame in Fig. 3A, labeled 3B1) also contained several enzymes (marked by a black arrow in Fig. 3B1 and listed in Table 1) in positions that did not vary substantially between the experimental groups. One spot (framed by a black circle) was strikingly only present in hypertensive samples (Fig. 3B1), and was absent in all of the other experimental groups in normotensive animals (Fig. 3B2) a, animals with iridectomy (Fig. 3B3), and those treated with Ti (Fig. 3B3, B4), Ti/Tr (Fig. 3B5), Ti/D (Fig. 3B6), and Ti/B (Fig. 3B7). Subsequent MALDI-MS confirmed that the spot corresponded to cryβb2. Cryβb3 (framed by a circle) was present equally in all groups (Fig. 3B1–B7), demonstrating the reproducibility of the applied method.

**Table 1:** Retinal proteins identified by two-dimensional gel electrophoresis and subsequent matrix-assisted laser desorption ionization mass spectrometry.

No.	Protein	Potential function	MW/kDA
1	Glucose related-protein 78	Cytosceleton	78
2	HSP 70 isoform 2	Protein folding ATPase activity	53
3	Glucose related-protein 75	Molecular chaperone	73,6
4	Glial fibrillary acidic protein	Cytosceleton	50
5	Enolase 2	Carbohydrate transport, metabolism	47
6	ATP synthase beta subunit	ATP biosynthesis	50,7
7	Beta actin	Structural protein,cytoskeleton	41,7
8	Retinaldehyde-binding protein	Transport of retinalaldehyde	44,5
9	Kinase associated HSP 90	Molecular chaperone	44,4
10	Crystalline mu	Amino acid transport, metabolism	33,5
11	Glucose-6-phosphatase isomerase	Carbohydrate transport, metabolism	29
12	Retinoidacid receptor responder protein	Type 2 membran protein	29
13	Recoverin	Regulation of rhodopsin	23
14	Class I beta tubulin	Tubulin, cytoskeleton	45
15	Synthaxin 2	Epithelial morphogenesis	33,3
16	Phosphatydylethanolamin bindingprotein	Lipid and ATP binding	20,7
17	HSP 60	Protein turnover, chaperonin	61
18	Enolase 1	Glycolysis, lyase	47
19	Craniofacial dev. Protein 1, cyclin G1	Cytochrome, craniofacial development	34
20	Calmodulin	Calcium-binding protein	35
21	Adaptor related protein complex3	Intracellular trafficking and secretion	34
22	Malate dehydrogenase	Energy production and conversion	36
23	Proteasome	Inhibitor of apoptosis	30
24	Peroxiredoxin 6	Thiol-specific antioxidant protein	25
25	ATP synthase delta subunit	ATP biosynthesis	19
26	Nucleoside diphosphate kinase B	Synthesis of nucleoside triphosphate	18
27	Carbonic anhydrase 1	Hydration of carbon dioxide	28
28	Triose-phtosphat isomerase	Glycolysis	28
29	βb3 crystallin	Structural protein of the eye lens	24
30	Phosphoglycerate mutase	Glycolysis	29
31	Bb2 crystallin	Structural protein of the eye lens	23
32	Acetyl-coenzyme A dehydrogenase	Fatty acid, lipid metabolism	24

**Figure 4:**
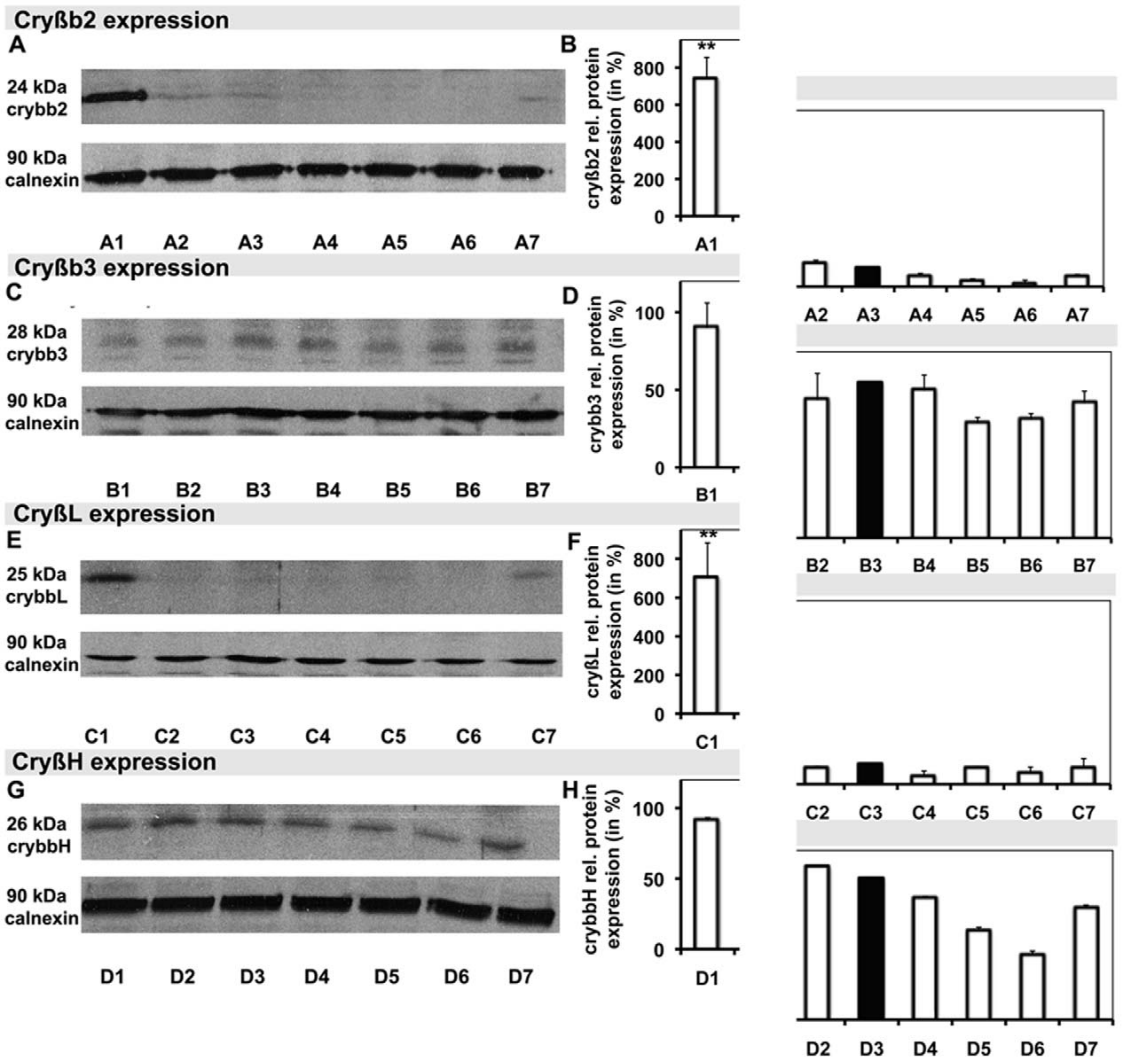
Specific Western blot analysis and the correlated graph of the relative density of selected proteins, including the application controls. A–G Blots of cryβb2 (**A**) cryβb3 (**C**), cryβL (**E**), and cryβH (**G**), and their correlated relative densities (**B**, **D**, **F**, and **H**, respectively), each with the corresponding control with calnexin. Hypertensive samples are demonstrated in **A1**–**D1**, normotensive samples in **A2**–**D2** and **A3**–**D3**, samples following Ti/B treatment in **A4**–**D4**, samples following Ti treatment in **A5**–**D5**, samples following Ti/Tr treatment in **A6**–**D6**, and samples following Ti/D treatment in **A7**–**D7**.

The numbers in column 1 correspond to those given in Fig. 4. Column 4 lists the molecular mass of the respective protein. HSP = heat-shock protein.

### Confirmation with Western blotting and immunohistochemistry

**Figure 5:**
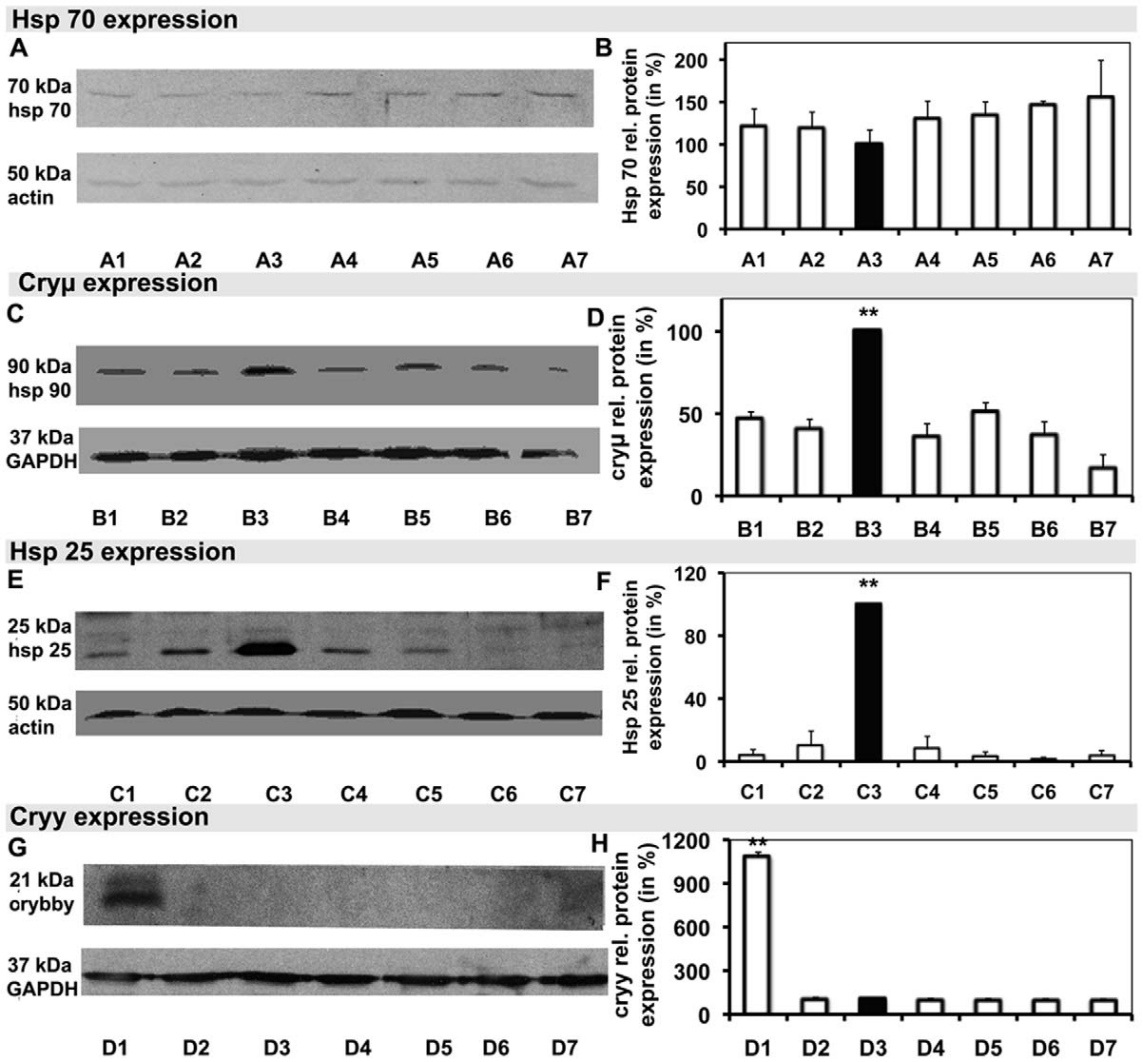
Specific Western blot analysis of selected proteins with the corresponding application controls. The graphs next to them illustrate the relative protein densities. **A**–**H** Blots of heat-shock protein (HSP)-70 (**A**), HSP-90 (**C**), HSP-25 (**E**), and cryγ (**G**) and their relative densities (**B**, **D**, **F**, and **H**, respectively). Hypertensive samples are shown in **A1**–**D1**, normotensive samples in **A2**–**D2** and **A3**–**D3**, samples following Ti/B treatment in **A4**–**D4**, samples following Ti therapy in **A5**–**D5**, samples following Ti/Tr treatment in **A6**–**D6**, and samples following Ti/D treatment in **A7**–**D7**.

Additional WB was performed for cryβb2, cryβb3, cryβL, cryβH, cryγ, cryμ, HSP-25, and HSP-70 on retinal samples to better characterize the changes in crystallin and HSP expression as seen through 2DE and MALDI-MS. Cryβb2, with a molecular mass of 23 kDa, was markedly up-regulated in the hypertensive retina (7.4±1.1-fold; *p*<0.001), being only marginally present in the normotensive retina (1.30±0.12-fold), and nearly totally absent in retinal samples treated with Ti (35.00±0.07-fold), Ti/Tr (20.0±0.1-fold), Ti/D (60.0±0.4-fold), and Ti/B (61.0±0.1-fold; Fig. 4A, B). Cryβb3 expression did not differ significantly among the groups (Fig. 4C, D). Consistent with cryβb2, cryβL was strongly expressed in hypertensive samples (7.2±1.8-fold; *p*<0.001), while it was only slightly expressed in all other groups (Fig. 4E, F). There were no marked changes in the expressions of either cryβH (Fig. 4G, H) or HSP-70 (Fig. 5A, B). Cryμ (Fig. 5C, D) and HSP-25 (Fig. 5E, F) were the most strongly expressed in the normotensive samples (*p*<0.001). Cryγ expression resembled the expressions of cryβb2 and cryβL, showing markedly higher expression in hypertensive samples (10.80±0.35-fold; *p*<0.001) than in the other groups (Fig. 5G, H).

**Figure 6:**
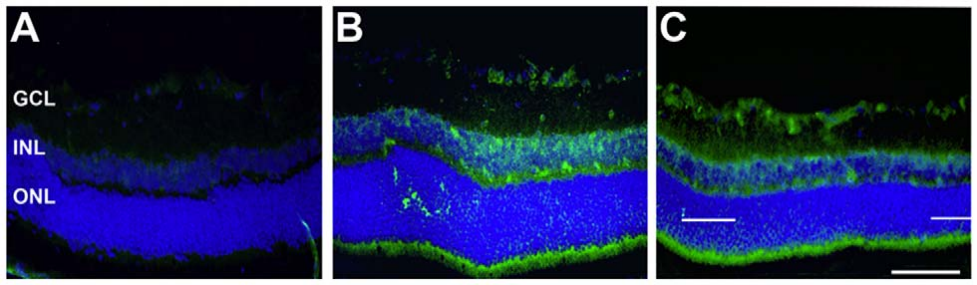
Immunohistochemical analysis of crystallins. Staining for cryβb (green) in a normotensive, sham-treated group (**A**), 7 days after induction of elevated IOP (**B**), and 28 days after induction of elevated IOP (**C**) in retinal slices. Cryβb staining revealed a distinctive up-regulation of cryβb after IOP elevation (**B**, **C**) relative to normotensive samples. Moreover, cryβb expression increased within the period of exposure to elevated IOP. The signal was more intense after 28 days (**C**) than after 7 days (**B**) of IOP elevation. Cryβb2 expression appeared predominantly in the RGC layer. Scale bar: 100 µm.

To confirm and visualize cryβb up-regulation within the retinal tissue, immunohistochemical staining was performed on normotensive and hypertensive retinal slices. IHC revealed up-regulation of cryβb after IOP elevation relative to normotensive samples (Fig. 6A, B). The signal increased with the duration of exposure to elevated IOP (Fig. 6C). Cryβb signaling was higher at 28 days than at 7 days after IOP elevation, and cryβb expression was localized predominantly in the RGC layer of retinal slices (Fig. 6B, C), indicating that RGCs are mainly adversely affected by elevated IOP in glaucoma.

### Microarrays and quantitative real-time polymerase chain reaction

**Figure 7:**
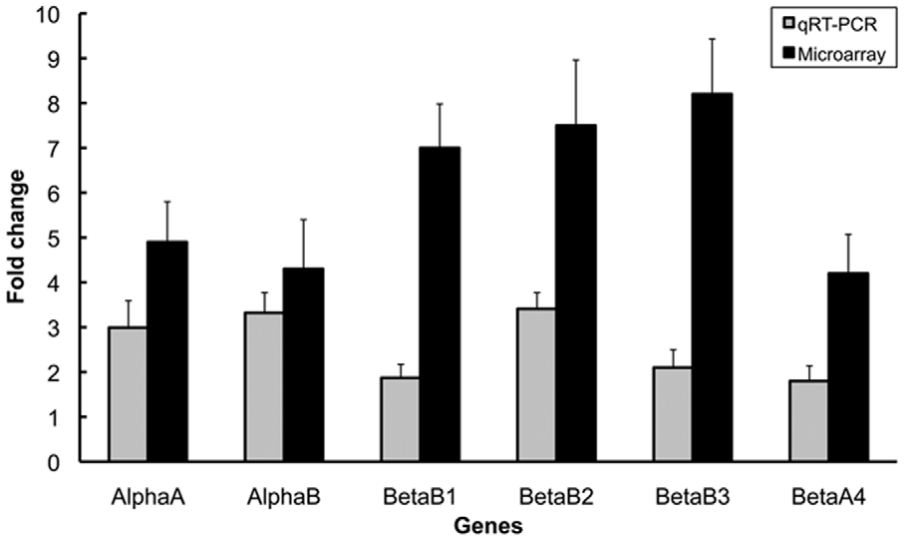
Confirmation of crystallin expression at the gene level by microarray analysis and subsequent quantitative real-time polymerase chain reaction. CryαA, cryαB, cryβb1, cryβb2, cryβb3, and cryβb4 were up-regulated due to elevated IOP at 8 weeks after IOP induction. The change **in** gene expression is expressed as the change relative to normotensive sham-treated retinas. The alterations in regulation of all six genes were statistically significant (*p*<0.001).

To confirm that the change in the expression of crystallin is reflected at the mRNA level, microarray analysis and additional qRT-PCR were performed on normotensive samples as well as on samples at 4 weeks after IOP elevation. Harvesting of the retinal samples 4 weeks after IOP elevation (before the initiation of antihypertensive therapy) enabled us to detect whether crystallin mRNAs were up-regulated due to IOP and down-regulated due to antihypertensive treatment. Microarray analysis and qRT-PCR were conducted after reverse transcription of isolated RNA for cryαA, cryαB, cryβb1, cryβb2, cryβb3, and cryβb4. Compared with normotensive retinas, the gene activity in hypertensive retinas was up-regulated by four- to tenfold (Fig. 7). All data support the initial hypothesis that retinal crystallins, and in particular cryβb2, are sensitive markers for detecting the pharmacological influences of drugs that are topically applied to reduce elevated IOP.

## Discussion

There are three principal findings from this study:

1. Prolonged IOP elevation modulates small HSPs, and in particular the pattern of expression of crystallin in the retina.
2. Pharmacological hypotensive treatments are effective at lowering IOP and consistently affect retinal cell metabolism including the regulation of distinctive crystallins to below baseline levels.
3. The beneficial effects on RGC survival of Ti/B, Ti/D, and Ti/Tr seem to operate independently of crystallin regulation.

Elevated IOP plays a major role in RGC apoptosis, and lowering of IOP remains the mainstay of glaucoma treatment [2]. Reducing IOP often helps to slow the progression of degenerative changes in glaucoma. RGC loss may proceed despite normalization of IOP following effective IOP reduction and the absence of elevated IOP beforehand [3], [5]. However, although elevated IOP is believed to make important contributions to optic-nerve and RGC damage, it is not the only risk factor involved, implying that further immunomodulatory and vascular factors are also crucial [5]. This finding has led to increasing interest in neuroprotective approaches.

Expanding on previous studies, we found that sustained elevation of IOP was correlated with changes in HSP expression. This may not be surprising since elevations in IOP have been shown to drive toxic metabolic changes within the retina, initiating a self-propagating vicious circle of RGC degeneration [10], ultimately culminating in apoptosis [4]. There are significant positive correlations between RGC loss and change in IOP [32] and duration of elevated IOP [33], and IOP elevation can directly induce RGC death by apoptosis. RGC death after exposure to elevated IOP seems to take place in two phases: direct IOP-dependent RGC apoptosis followed by a second, slower phase involving neuron loss due to toxic and inflammatory effects of the primary degenerating neurons [31]. Inhibition of this second IOP-triggered self-propagating process of RGC degeneration may lead to new therapeutic approaches. Regulation of HSPs appears to reflect cellular attempts to resist an abnormal IOP.

To further scrutinize the molecular cascades initiated by elevations and reductions in IOP, we adopted the clinically well-proven therapy of topical application of IOP-lowering drugs. Daily topical medication is routinely performed in the clinical treatment of patients, and this produces crucial intraretinal responses that can be detected with sophisticated proteomic and genomic methods. We used an experimental animal model as a surrogate of glaucoma to detect the anticipated glaucomatous changes [18], [20]. Our data showed the IOP-dependent regulation of small HSPs and crystallins at both the proteomic and mRNA levels. HSPs are a family of cellular chaperones that are defined according to their molecular masses (in kDa) as HSP-60, HSP-70, HSP-90, and small HSPs (a group with a molecular mass of 20–30 kDa).

Closely related in sequence, and subsummarized to the ubiquitous HSPs, are the crystallins, some of which display partial chaperoning functions [34]. Crystallins have long been considered as the structural proteins of the vertebrate lens [35], and in particular are synergistically responsible for refractive functions such as the preservation of lens transparency throughout life [36]. Crystallins have been localized in the nervous system and the retina, leading to advanced interest in their functions in extralenticular tissues [34], [37]–[38].

The ubiquitous occurrence of crystallins in several tissues and cell types (including RGCs), and their homology and close relationship with the ubiquitous HSPs have led some of them to be classified as stress proteins, although they are also vital to normal tissue differentiation [36], [37]. In this context, it is believed that the crystallins are temporarily differentially expressed within the rat retina after various forms of injury [12], [39] indicating their involvement both in injury and in postinjury repair. For instance, the expression of cryαB is increased in various neurological disorders [40] such as Alexander's disease [41], Creutzfeld-Jakob disease, and Parkinson's disease [42]. The up-regulation of cryα, cryβ, and cryγ in the retina has been found consistently in gene expression studies after ischemia–reperfusion injury [43], light injury [44], and retinal tears [39], and in diabetic rats [45]. Crystallin regulation has recently been reported at the mRNA and protein levels in both hereditary and experimental models of glaucoma [12], [19]. Cryβb expression is increased in the glaucomatous optic nerves of monkeys [46]. Interestingly, crystallin expression patterns shift due to the period of exposure to elevated IOP, exhibiting down-regulation of crystallins at the mRNA level and up-regulation to control levels at 2 and 5 weeks after IOP elevation, respectively. It is assumed that crystallin transcription may be stimulated throughout RGC degeneration in response to IOP elevation or in response to the dynamics of elevated IOP, independent of RGC degeneration [12]. According to these findings, the marked up-regulation of crystallin mRNA and protein after IOP elevation and the subsequent down-regulation following antihypertensive treatment reflects the IOP-dependent regulation of crystallins.

According to our results, cryβb2 is expressed mainly in the RGCs, as presumed previously [12]. Three crystallins (cryβb2, cryβbL, and cryβbγ) were strikingly expressed in hypertensive samples compared to normotensive controls, and down-regulated to and below baseline levels following effective hypotensive treatment. On the other hand, the expressions of cryβb3, cryβbH, and HSP-70 remained unchanged, and those of cryμ and HSP-25 were significantly higher in normotensive samples, to become down-regulated after IOP elevation, and to remain down-regulated despite effective IOP lowering.

In addition to acting within neurons, HSPs induce immunomodulatory cascades in glaucoma [16]. Titers of circulating antibodies against small HSPs are increased in the serum of glaucoma patients. Moreover, HSPs are considered to be associated with and responsible for increased RGC death. The functions of the immune system in glaucoma are probably surveillance and regulation, in which signaling pathways of the immune system regulate cell death in response to conditions that stressRGCs, such as elevated IOP or factors produced as a consequence thereof [47]. Whether those antibodies are produced primarily as autoantibodies or are released in response to enhanced expression of small HSPs due to elevated IOP remains unclear, since HSPs are known to have strong antigenetic potential [48]–[49]. The latter mechanism would require the release of cryβb into the plasma serum to induce an antigen reaction, which seems to be the case, at least for cryβb2. Cryβb2 can be released out of the cells into the culture medium and can be taken up by the cells again. Therefore crybβ2 presents as a molecule that trafficks between the cytosol and the extracellular space [13].

We found a drug-specific regulation of the pattern of crystallin expression and neuroprotective effects of antihypertensive treatments with Ti/Tr, Ti/D, and Ti/B that appear to be independent of each other. The drug components used in this study are assumed to be neuroprotective in various experiments, and the mechanisms involved have been established. In a manner unrelated to their β-adrenoreceptor blocking activity [50], β-adrenergic agonists reduce ligand-stimulated calcium and sodium influx into cells through direct interaction with L-type voltage-dependent calcium channels [51] and voltage-sensitive sodium channels [52]. α-2a agonists seem to inhibit glutamate and aspartate accumulation [53], up-regulate antiapoptotic genes such as *bcl-2* and *bcl-xl*, and produce neurotrophic factors, most evidently mediated through α-2a adrenoreceptor activation [54]. Prostaglandin F_2α_ analogues exert their neuroprotective effects via the retinal prostaglandin F receptor [55] by reducing the release of lactate dehydrogenase and through p44/p42 mitogen-activated protein kinase and caspase-3 inhibition [25]. Carbonic anhydrase inhibitors work by augmenting retrobulbar blood flow in glaucoma patients [56]. Our data show that antihypertensive treatment induces retinal metabolic changes and effectively reduces stress to neurons, as seen strikingly through the down-regulation of various crystallins.

In conclusion, our study shows that elevated IOP causes alterations at both the histopathological and proteomic levels, in accordance with previous reports. The novel findings of our study are the changes in the pattern of crystallin expression. We have also shown that antihypertensive treatment reverses specific IOP-induced alterations within the retina at the proteomic level. This effect is independent of the neuroprotective effects observed in our *in vivo* model, suggesting that the eye drops exert a direct effect on retinal metabolism. The significance of the marked regulation of small HSPs and crystallins, in particular due to neuronal degeneration following elevated IOP and antihypertensive treatment, merits further investigation.
